# Robust optimization in lung treatment plans accounting for geometric uncertainty

**DOI:** 10.1002/acm2.12291

**Published:** 2018-03-10

**Authors:** Xin Zhang, Yi Rong, Steven Morrill, Jian Fang, Ganesh Narayanasamy, Edvaldo Galhardo, Sanjay Maraboyina, Christopher Croft, Fen xia, Jose Penagaricano

**Affiliations:** ^1^ Department of Radiation Oncology University of Arkansas for Medical Science Little Rock AR USA; ^2^ Department of Radiation Oncology University of California at Davis Comprehensive Cancer Center Sacramento CA USA

**Keywords:** dosimetric comparison, RayStation, robust optimization, treatment planning system

## Abstract

Robust optimization generates scenario‐based plans by a minimax optimization method to find optimal scenario for the trade‐off between target coverage robustness and organ‐at‐risk (OAR) sparing. In this study, 20 lung cancer patients with tumors located at various anatomical regions within the lungs were selected and robust optimization photon treatment plans including intensity modulated radiotherapy (IMRT) and volumetric modulated arc therapy (VMAT) plans were generated. The plan robustness was analyzed using perturbed doses with setup error boundary of ±3 mm in anterior/posterior (AP), ±3 mm in left/right (LR), and ±5 mm in inferior/superior (IS) directions from isocenter. Perturbed doses for D_99_, D_98_, and D_95_ were computed from six shifted isocenter plans to evaluate plan robustness. Dosimetric study was performed to compare the internal target volume‐based robust optimization plans (ITV‐IMRT and ITV‐VMAT) and conventional PTV margin‐based plans (PTV‐IMRT and PTV‐VMAT). The dosimetric comparison parameters were: ITV target mean dose (D_mean_), R_95_(D_95_/D_prescription_), Paddick's conformity index (CI), homogeneity index (HI), monitor unit (MU), and OAR doses including lung (D_mean_, V_20 Gy_ and V_15 Gy_), chest wall, heart, esophagus, and maximum cord doses. A comparison of optimization results showed the robust optimization plan had better ITV dose coverage, better CI, worse HI, and lower OAR doses than conventional PTV margin‐based plans. Plan robustness evaluation showed that the perturbed doses of D_99_, D_98_, and D_95_ were all satisfied at least 99% of the ITV to received 95% of prescription doses. It was also observed that PTV margin‐based plans had higher MU than robust optimization plans. The results also showed robust optimization can generate plans that offer increased OAR sparing, especially for normal lungs and OARs near or abutting the target. Weak correlation was found between normal lung dose and target size, and no other correlation was observed in this study.

## 1 INTRODUCTION

Robust optimization is primarily used to plan intensity modulated proton therapy (IMPT) 1 and it was not until recently that robust optimization techniques have been available for x‐ray beam in radiation therapy treatment planning system.^2^ Robust optimization methods have been used in radiation therapy to account for position uncertainties relative to the target volume during treatment delivery. Position uncertainties come from two sources: tumor motion and variations in tumor shapes, and patient setup uncertainties. One way to approach these uncertainties is to use minimax optimization.^3^ Instead of expanding the internal target volume (ITV) with a fixed margin to create the planning target volume (PTV), robust optimization allows entering the setup uncertainties into the planning computer and discretizes them into multiple scenarios (shifts within the margin bounds). The minimax optimization method minimizes the objective function such that the prescription holds true even in the worst case scenario. That is, robust optimization method generates scenario‐based plans that have plan quality considered at least equivalent to static PTV margin‐based plans.^4^, ^5^, ^6^ This method has the potential to reduce the doses to healthy tissues, especially for tumors with substantially larger intrafraction motions in which there could be overlap between PTV and organ at risk (OAR).

The purpose of this study was to investigate the use of RayStation (v.5.4, RaySearch Laboratories, Sweden) photon robust optimization method for planning lung cancer patients. Both robust‐optimized intensity modulated radiotherapy plans (ITV‐IMRT) and robust‐optimized volumetric modulated arc therapy plans (ITV‐VMAT) were evaluated. A dosimetric study was performed to compare robust optimization plans with the corresponding traditional PTV margin‐based plans (PTV‐IMRT and PTV‐VMAT). A correlation study was also performed to investigate the relationship between the target mean dose and the tumor size or tumor motion and the relationship between normal lung dose and tumor size or tumor motion.

## 2 MATERIALS AND METHODS

### 2.A Patient characteristics

Twenty lung cancer patients who were previously treated in our clinic were selected. Tumors located at various anatomical regions within the lungs were selected (11 in upper lobe, 8 in lower lobe, and 1 in middle lobe). Among these, 4 were centrally located and 16 were peripherally located according to the definition of peripheral or central tumor location defined in RTOG 0915.^7^ The median ITV volume was 10.39 cc (3.29–107.23 cc) and the median PTV volume was 38.57 cc (19.12–210.23 cc). The tumor motion was determined by measuring the largest shifted distance from the center of tumor mass in the inferior–superior (IS), left–right (LR), and anterior–posterior (AP) directions. The isocenter was placed automatically at the center of tumor mass during treatment planning and the tumor motion was measured based on 4D‐CT volume image set. The median maximum tumor motion within 3D mobility vectors was 1.45 cm (0.54–3.4 cm). The patient characteristic details are listed in Table 1.

**Table 1:** Patient characteristics

Patient #	ITV (cc)	PTV (cc)	Tumor a location	Tumor motion (cm)
1	43.99	126.26	LUL‐p	0.60
2	8.52	31.40	RUL‐p	2.59
3	5.69	23.84	LLL‐p	2.50
4	17.90	53.34	RUL‐c	3.07
5	28.03	78.61	RUL‐c	3.40
6	6.13	24.18	LUL‐ p	2.12
7	10.40	41.01	RLL‐p	2.64
8	3.29	19.12	LUL‐p	1.00
9	45.59	111.36	RLL‐p	2.4
10	9.90	33.26	LLL‐p	1.45
11	9.88	35.23	RUL‐c	0.70
12	10.37	36.12	LLL‐p	2.00
13	3.88	21.40	LUL‐p	0.70
14	25.20	67.55	RUL‐p	1.07
15	26.20	82.20	LUL‐p	0.54
16	7.05	30.70	LLL‐p	1.15
17	6.28	27.50	RUL‐p	1.20
18	18.69	66.78	RML‐p	1.00
19	107.23	210.23	RLL‐c	1.87
20	38.38	94.23	LLL‐p	2.37
Median (range)	10.39 (3.29–107.23)	38.57 (19.12–210.23)		1.45 (0.54–3.4)

a LUL, left upper lobe; LLL, left lower lobe; RUL, right upper lobe, RML, right middle lobe; RLL, right lower lobe, c, central; p, peripheral.

### 2.B ITV and PTV contour generation

To account for potential lung tumor motion for each patient, four‐dimensional CT (4DCT) images with ten respiratory phases (0% to 90%) were acquired on a CT‐simulator (Brilliance CT Big Bore, Philips, Cleveland, OH, USA) for each patient and imported to the RayStation treatment planning system (TPS). Ten gross tumor volumes (GTV) corresponding to different phase image datasets (GTV 0% to GTV 90%) were identified and manually delineated by the treating radiation oncologist. A single planning ITV contour was then created by encompassing all ten‐phases of GTVs. The PTV contour was generated by extending the ITV contour to 0.5 cm in LR and AP direction and 1.0 cm along IS direction.

The robust treatment plans were generated based on all ten respiratory phases of the CT image datasets to evaluate the plan robustness caused by the tumor motion. Plan comparison between robust optimization and PTV margin‐based optimization was performed using treatment plans generated based on the 20% phase of the CT image dataset.

### 2.C ITV robust optimization plan and PTV margin‐based plan

ITV robust optimization plans were generated using minimax optimization method by minimizing the penalty of the worst case scenario. The minimax method does not minimize the worst of all possible scenarios, but the worst scenario within some predefined range. It considers only scenarios that are physically reliable; with unnecessary conservative case scenarios being avoided.^3^, ^4^ The range of patient setup error specified by the user was 0.5 cm in LR, 1.0 cm in IS, and 0.5 cm in AP direction for this study. This range of uncertainty was selected based on RTOG 0236 and 0915 protocols.^8^, ^9^ The total numbers of scenarios considered in the minimax optimization are related to the size of the uncertainty range specified in the TPS. In this study, a total of seventeen scenarios were generated based on the selected uncertainty.

Four treatment plans were generated per patient, among them two plans were robust optimization plans (ITV‐IMRT and ITV‐VMAT) and two plans were PTV margin‐based plans (PTV‐IMRT and PTV‐VMAT). For comparison purposes, the planning and optimization parameters were kept identical for all plans except those in ITV optimization‐based plans where the robust optimization function was used. The prescription dose (D_p_) was such that at least 95% of the ITV or PTV receives 60 Gy in eight fractions. The lung doses were constrained as V_20 Gy_<20% and V_15 Gy_<37%.^10^

### 2.D Plan evaluation

Plan robustness was evaluated by calculating perturbed doses from multiple spatially shifted 3D vectors in both positive and negative directions. The shifted values from isocenter were 3 mm in AP direction (0, 0, ±3), 3 mm in LR direction (±3, 0, 0), and 5 mm in SI direction (0, ±5, 0). A total of six perturbed dose distributions were computed for each plan. Evaluation metrics included perturbed doses of D_99_ (isodose that cover 99% of ITV), D_98_ (isodose that cover 98% of the ITV), and D_95_ (isodose that cover 95% of the ITV). The perturbed doses were computed for each of the six shifted isocenter plans.

Other dosimetric comparisons and evaluations performed for both robust optimization and conventional margin‐based plans included: ITV target mean dose (D_mean_), R_95_ (D_95_/D_p_), Paddick's conformity index, homogeneity index*,* MU, and OAR doses including normal lung (D_mean_,V_20 Gy_ and V_15 Gy_), chest wall, heart, esophagus, and maximum cord doses. The Paddick's conformity index (CI) is calculated based on the following equation:

$$CI=\frac{TV_{\mathrm{PIV}}^{2}}{TV\times V_{\mathrm{PIV}}}$$

Where *TV* is the target volume, *TV*_*PIV*_ is the target volume covered by the prescription isodose volume (PIV), and *V*_*PIV*_ is the total prescription isodose volume. The homogeneity index (HI) is calculated based on the following equation:

$$HI=\frac{D_{2\%}-D_{98\%}}{D_{p}}$$

Where D_p_ is the prescription dose.

### 2.E Statistics

A multivariate ANOVA statistical test^11^ was performed to compare the statistical difference between multigroup treatment plans (ITV‐IMRT, ITV‐VMAT, PTV‐IMRT, and PTV‐VMAT). For the robust optimization plans, a Student t‐test was performed to compare the pairwise difference between two treatment techniques (ITV‐IMRT and ITV‐VMAT) for robust optimization plans.^12^ A *P*‐value <0.05 was considered statistically significant. A study was performed using a linear model with *SigmaPlot* software version (v.13, Systat Software, Inc., San Jose, CA, USA, http://www.systatsoftware.com) to evaluate the correlation between the target mean dose and the perturbed dose of D_99_, D_98_, and D_95_ versus the tumor size/tumor motion. Similarly, a study was performed to evaluate the correlation between the normal lung tissue doses versus the tumor size, and tumor motion. In addition, correlation was estimated between ITV mean dose and tumor size, tumor motion.

## 3 RESULTS

### 3.A Target dose evaluation

The median and ranges of target mean doses (D_mean_), target coverage (R_95_), HI, CI, and MU from robust optimization and PTV margin‐based plans using both IMRT and VMAT treatment techniques are shown in Table 2.

**Table 2:** Median and ranges of D_mean_, R_95_, HI, CI, and MU for ITV target using IMRT and VMAT technique

	IMRT			VMAT		
	ITV robust^a^	PTV nonrobust^b^	ANOVA test Significant?	ITV robust^a^	PTV nonrobust^b^	ANOVA test Significant?
D_mean_ (Gy)	61.85 (61.05–66.43)	62.76 (61.21–83.3)	Yes *P* = 0.012	64.44 (61.32–67.11)	65.77 (63.14–82.48)	Yes *P* = 0.002
R_95_	1.00 (0.99–1.01)	1.03 (1.01–1.32)	Yes *P* < 0.001	1.00 (1.00–1.00)	1.06 (1.03–1.29)	Yes *P* < 0.001
HI	0.073 (0.034–0.205)	0.040 (0.022–0.163)	Yes *P* < 0.001	0.124 (0.042–0.246)	0.069 (0.04–0.206)	Yes *P* < 0.001
CI	0.60 (0.31–0.82)	0.26 (0.03–0.50)	Yes *P* < 0.001	0.57 (0.32–0.76)	0.26 (0.12–0.50)	Yes *P* < 0.001
MU	1365 (1091–2691)	1844 (1188–2960)	Yes *P* < 0.001	1200 (1046–1867)	1503 (1124–2194)	Yes *P* < 0.001

a ITV‐based robust optimization plans.

b PTV margin‐based optimization plans.

**Figure 1:**
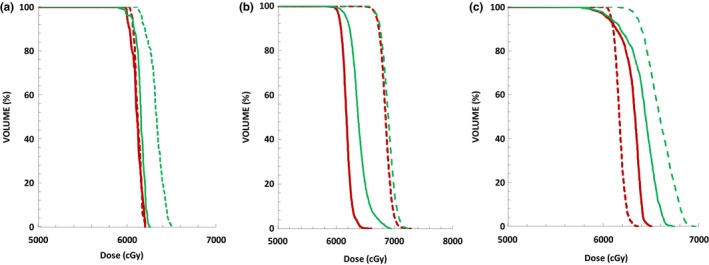
ITV DVHs for robust‐optimized IMRT plan (solid red line), robust‐optimized VMAT plans (solid green line), PTV margin‐based IMRT plan (dotted red line), and PTV margin‐based VMAT plan (dotted green line). (a) Pt8, max target motion d = 1 cm, ITV = 3.29 cc. (b) Pt19, max target motion d = 1.87 cm, ITV = 107.23 cc. (c) Pt5, max target motion d = 3.4 cm, ITV = 28.03 cc.

Table 2 shows that the ITV target dose coverage satisfied the prescription requirement for both robust optimization and PTV margin‐based plans. In addition, the robust optimization plans showed better CI and worse HI compared to PTV margin‐based plans. Figure 1 shows examples of ITV DVHs for three patients with different tumor sizes and tumor motion distances for robust optimization (solid line) and PTV margin‐based (dotted line) IMRT and VMAT plans.

The differences between the IMRT and VMAT technique using robust optimization method were compared and the results are shown in Table 3 and Fig. 1. It was observed that there was no statistical difference for ITV dose coverage and that there was a statistically significant difference with better HI and CI for ITV‐IMRT compared to ITV‐VMAT plans.

**Table 3:** Median and ranges of D_mean_, R_95_, HI, CI, and MU for ITV target from robust optimization plans

	D_mean_(Gy)	R_95_	HI	CI	MU
ITV‐IMRT^a^	61.85 (61.05–66.43)	1.00 (0.99–1.01)	0.073 (0.034–0.205)	0.60 (0.31–0.82)	1365 (1091–2691)
ITV‐VMAT^b^	64.44 (61.32–67.11)	1.00 (1.00–1.00)	0.124 (0.042–0.246)	0.57 (0.32–0.76)	1200 (1046–1867)
Student *t*‐test significant?	Yes *P <* 0.001	No *P* > 0.05	Yes *P* < 0.001	Yes *P* = 0.004	Yes *P* = 0.003

a robust optimization ITV‐IMRT plan.

b robust optimization ITV‐VMAT plan.

Perturbed doses were calculated to evaluate plan robustness. ITV perturbed median doses and ranges of D_99_, D_98_, and D_95_ calculated from six shifted isocenter plans are shown in Tables 4, 5, 6. Results show that the ITV perturbed doses of D_99_, D_98_ and D_95_ were covered at least more than 95%, 96%, and 98.5% of the prescribed dose for all six shifted isocenter plans.

**Table 4:** ITV perturbed doses of D_99_ (Gy)

D (mm)^a^	(−3,0,0)	(3,0,0)	(0,−5,0)	(0,5,0)	(0,0,−3)	(0,0,3)
ITV‐IMRT	58.57 (49.58–59.68)	58.38 (50.44–59.56)	57.93 (50.81–59.42)	58.18 (51.74‐59.14)	58.28 (48.05‐59.70)	58.61 (50.98‐59.29)
ITV‐VMAT	57.15 (49.81–59.68)	56.81 (50.51–59.49)	56.66 (50.40–59.42)	57.03 (50.01‐58.80)	56.97 (47.26‐59.48)	57.08 (51.1‐59.05)
Student *t*‐test significant?	No *P* > 0.05	Yes *P* = 0.016	Yes *P* = 0.002	Yes *P* = 0.002	Yes *P* = 0.008	Yes *P* = 0.028

a shifted distance in mm from isocenter in (±x, ±y, ±z) direction.

**Table 5:** ITV perturbed doses D_98_ (Gy)

D (mm)^a^	(−3,0,0)	(3,0,0)	(0,−5,0)	(0,5,0)	(0,0,−3)	(0,0,3)
ITV‐IMRT	58.57 (52.8–59.8)	58.38 (53.24–59.86)	57.93 (52.82–59.87)	58.18 (54.31–59.58)	58.28 (52.71–59.9)	58.61 (53.4–59.45)
ITV‐VMAT	58.25 (52.84–59.95)	57.72 (53.74–59.8)	57.43 (52.82–59.51)	57.94 (53.24–59.12)	57.84 (51.9–59.93)	57.89 (53.72–59.35)
Student *t*‐test significant?	No *P* > 0.05	No *P* > 0.05	Yes *P* = 0.001	Yes *P* = 0.012	Yes *P* = 0.015	Yes *P* = 0.026

a shifted distance in mm from isocenter in (±x, ±y, ±z) direction.

**Table 6:** ITV perturbed doses of D_95_ (Gy)

D (mm)^a^	(−3,0,0)	(3,0,0)	(0,−5,0)	(0,5,0)	(0,0,−3)	(0,0,3)
ITV‐IMRT	59.52 (56.71–60.07)	59.4 (57.62–60.08)	59.2 (56.41–60.13)	59.19 (56.34–60)	59.47 (58–60.15)	59.4 (57.7–60.26)
ITV‐VMAT	59.47 (56.26–60.6)	59.05 (56.71–60.58)	58.85 (55.07–60.28)	58.94 (55.26–59.88)	59.17 (56.84–60.7)	59.18 (58.05–60.35)
Student *t*‐test significant?	No *P* > 0.05	No *P* > 0.05	Yes *P* < 0.001	Yes *P* = 0.014	No *P* > 0.05	No *P* > 0.05

a shifted distance in mm from isocenter in (±x, ±y, ±z) direction.

A Student t‐test was performed to compare the difference between IMRT and VMAT treatment techniques for the plans using robust optimization methods (ITV‐IMRT vs. ITV‐VMAT). For the perturbed doses of D_99_ and D_98_, the results showed statistically significant differences between ITV‐IMRT and ITV‐VMAT plans in a majority of shifted points where ITV‐IMRT perturbed dose coverage was better than the coverage of ITV‐VMAT plans. For the perturbed doses of D_95_, except in IS direction, there was no statistical difference between all shifted plans. A statistical correlation test was performed and no correlation was observed between the perturbed doses (D_99_, D_98_, and D_95_) and the tumor volume, tumor motion.

### 3.B OAR doses

The median mean doses and ranges for lung, heart, esophagus, and median maximum doses and ranges for spinal cord from robust optimization and PTV margin‐based plans using IMRT and VMAT treatment techniques are shown in Table 7. Results showed statistically significant differences between robust optimization and PTV margin‐based plans. The robust optimization plans showed lower OARs doses compared to PTV margin‐based plans.

**Table 7:** Median and ranges of OAR doses (Gy) for robust optimization and PTV margin‐based IMRT and VMAT plans

IMRT	Lung			Heart (D_mean dose_)	Esophagus (D_mean_ _dose_)	Spinal cord (D_maximum dose_)
	D_mean dose_	V_20 Gy_ (%)	V_15 Gy_ (%)			
ITV‐IMRT^a^	3.42 (2.78–10.55)	4.69 (2.54–19.01)	6.58 (3.94–22.47)	1.62 (0.08–11.18)	2.19 (0.78–10.29)	10.26 (1.12–18.88)
PTV‐IMRT^b^	4.54 (3.14–12.80)	5.66 (3.15–20.6)	8.19 (5.18–23.96)	2.02 (0.08–13.44)	2.89 (0.94–12.06)	12.56 (1.56–21.59)
ANOVA *t*‐test significant?	Yes *P* < 0.001	Yes *P* < 0.001	Yes *P* < 0.001	Yes *P* = 0.002	Yes *P* < 0.001	Yes *P* < 0.001

VMAT	Lung			Heart (D_mean dose_)	Esophagus (D_mean dose_)	Spinal cord (D_maximum dose_)
	D_mean_(Gy)	V_20 Gy_ (%)	V_37_ (%)			
ITV‐VMAT^a^	3.6 (2.59–10.59)	4.6 (2.65–18.49)	6.78 (4.06–22.14)	1.60 (0.08–12.25)	1.91 (0.7–10.53)	9.39 (1.36–22.29)
PTV‐VMAT^b^	4.49 (3.08–12.83)	6.18 (3.29–20.37)	8.75 (4.89–24.01)	1.93 (0.10–14.64)	2.61 (0.86–11.61)	11.40 (1.69–23.95)
ANOVA *t*‐test significant?	Yes *P* < 0.001	Yes *P* < 0.001	Yes *P* < 0.001	Yes *P* = 0.003	Yes *P* = 0.006	Yes *P* < 0.001

a ITV‐based robust optimization plans.

b PTV margin‐based optimization plans.

Similar results of OAR doses for robust optimization (ITV‐IMRT and ITV‐VMAT) plans are shown in Table 8 and results showed no statistically significant differences between robust optimization ITV‐IMRT and ITV‐VMAT plans.

**Table 8:** Median and ranges of OAR doses (Gy) from robust optimization IMRT and VMAT plans

	Lung			Heart (D_mean dose_)	Esophagus (D_mean dose_)	Spinal cord (D_maximum dose_)
	D_mean_ _dose_	V_20 Gy_ (%)	V_37_ (%)			
ITV‐IMRT	3.42 (2.78–10.55)	4.69 (2.54–19.01)	6.58 (3.94–22.47)	1.62 (0.08–11.18)	2.19 (0.78–10.29)	10.26 (1.12–18.88)
ITV‐VMAT	3.6 (2.59–10.59)	4.60 (2.65–18.49)	6.78 (4.06–22.14)	1.60 (0.08–12.25)	1.91 (0.7–10.53)	9.39 (1.36–22.29)
Student *t*‐test significant?	No *P* > 0.05	No *P* > 0.05	No *P* > 0.05	No *P* > 0.05	No *P* > 0.05	No *P* > 0.05

A statistical correlation study was performed to evaluate the correlation between the normal lung tissue dose and tumor volume size/tumor motion, and results showed weak correction with the tumor volume size (*r* ^*2*^ = 0.138 for ITV‐IMRT plans, *r* ^*2*^ = 0.110 PTV‐IMRT plans, *r* ^*2*^ = 0.255 for ITV‐VMAT plans, and *r* ^*2*^ = 0.048 for PTV‐VMAT plans). Normal lung tissue dose showed a better correlation with tumor volume for robust optimization plans compared to PTV margin‐based plans. No correlation was found between normal lung tissue dose and tumor motion in this study.

## 4 DISCUSSION

The main goal of this study was to investigate the photon robust optimization method for lung cancer patient and to evaluate the plan robustness with perturbed doses shifted from isocenter. In addition, robust optimization plans were compared with traditional PTV margin‐based treatment plans for ITV dose coverage, HI, CI, MU and OAR doses. Instead of traditionally representing the target region using PTV, the setup and target position uncertainties were entered into the treatment planning system and optimized concomitantly with the dose optimization using the minimax optimization method.

**Figure 2:**
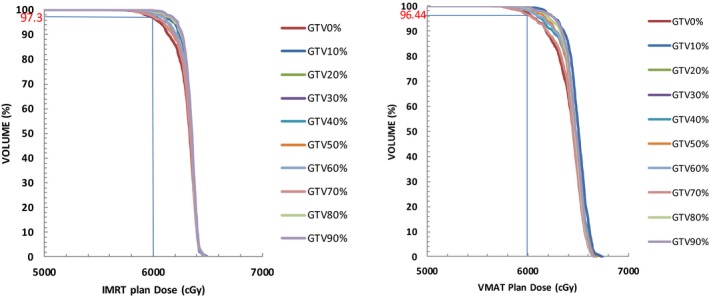
Example of target DVHs from ITV‐IMRT and ITV‐VMAT plans generated from ten respiratory phases (Pt5, max target motion d = 3.4 cm, ITV = 28.03 cc).

**Figure 3:**
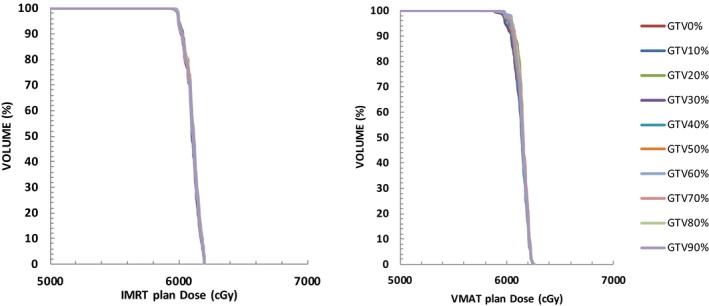
Example of target DVHs from ITV‐IMRT and ITV‐VMAT plans generated from ten respiratory phases (Pt8, max target motion d = 1 cm, ITV = 3.29 cc).

There are several important factors that can contribute to the deviation between planned and delivered dose. These factors include organ motion, geometrical uncertainties during target delineation and random/systematic errors during positioning and treatment.^13^ Organ motion and geometrical uncertainties will directly affect the definition of ITVs. Generally there are two methods to generate ITV; one is to manually contour GTV using ten selected phases of 4DCT datasets; the other is to generate an ITV contour based on the maximum intensity projections (MIP) that was automatically generated from 4DCT simulation. The former method is time consuming and requires more physician time. However, the latter was reported to produce smaller ITV volumes compared to the ten‐phase manually contouring method.^14^ Therefore in this study, we decided to use the former method to define ITV, with the additional benefit of obtaining dose distributions in each phase of the respiratory cycle. The ten‐phase manually contouring method is not very practical for the routine clinic performance. The simplified ITV contouring method needs to be developed such as using MIP plus two more image datasets (the full inspiration and expiration phases of the respiratory cycle). However, this proposed method need to be verified and it is beyond the scope of this study. For each patient, ten robust optimization treatment plans were generated corresponding to each of the ten‐phase image datasets. Differences were found in ITV dose distributions for both IMRT and VMAT treatment plans. Figs. 2 and 3 show an example of two patients’ ITV DVHs, whereas Fig. 2 shows the ten‐phase DVHs for one patient with the largest tumor motion among all 20 patients in this study. Figure 3 shows ten‐phases DVHs for the patient with the smallest target motion. It was noticed that the ten‐phase ITV dose distributions were more widely spatially distributed for the patients with larger tumor motions, while they almost overlapped with each other for patients with smaller tumor motions.

In comparing robust and nonrobust optimization plans, robust optimization plans had better ITV dose coverage, better CI, and worse HI compared to corresponding PTV margin‐based plans (Table 2 & Fig. 1) for both IMRT and VMAT techniques. This study showed that PTV margin‐based plans had higher mean doses and R_95_, with R_95_ values all larger than 1.0. The worse CI values in PTV margin‐based plans could be caused by larger *TV*_*PIV*_ (the target volume covered by the prescription isodose volume), which decreases the plan conformity. It was also noticed that PTV margin‐based plans had greater variations in D_mean_ and R_95_ compared to robust‐optimized plans. This could be explained by the following reasons: robust optimization use minimax optimization method to minimize the objective function, so the planning target volume tends to be smaller compared to the traditional PTV margin‐based planning target volume. In addition, when the planning target is large in size or adjacent to the surrounding critical organs (i.e., chest wall), it is more difficult for PTV margin‐based plan to reduce the critical target dose (chest wall or normal lung tissue) to an acceptable level. So PTV margin‐based plans might be compromised more which may result in a larger range of D_mean_ and R_95_ compared to robust‐optimized plan.

In robust optimization, a minimal margin is applied to the target.^15^ Therefore, it is also important to confirm adequate target dose coverage in evaluating plan robustness. This is even more important in moving targets. Perturbed dose was computed to evaluate plan robustness by specifying a shift from the isocenter in (LR, IS, AP) directions. Currently, there is no standard for perturbed dose computation. The shifted values used (±3 mm, ±5 mm, ±3 mm) in this study were based on empirical data from other published studies 16, 17 and from our clinical experience. Perturbed doses of D_99_, D_98_, and D_95_ were computed for all robust optimization plans and results showed that D_99_≥95% of D_p_, D_98_≥96.6% of D_p_, and D_95_≥98% of D_p_. A Student t‐test showed that the ITV dose coverage was worse for ITV‐VMAT plans compared to ITV‐IMRT plans in terms of D_99_ and D_98_. For perturbed dose of D_95_, no difference was found between ITV‐VMAT and ITV‐IMRT plans except for D_95_ in IS direction. This might indicate that VMAT plans are more sensitive for off isocenter uncertainty compared to IMRT plans.

**Figure 4:**
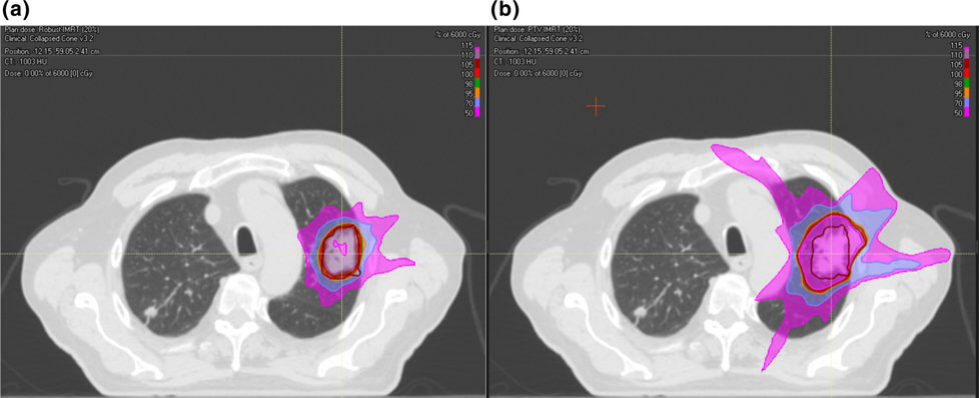
Isodose distribution from robust optimization IMRT and PTV margin‐based IMRT plans. (a: robust optimization ITV‐IMRT plan; b: PTV margin‐based PTV‐IMRT plan)

Robust optimization plans spared more of the OAR doses including lung, chest wall, heart, esophagus, and maximum cord doses compared to PTV margin‐based plans (Table 7). The robust optimization plans have the advantage of finding the best scenario for the trade‐off between target coverage robustness and OAR sparing. This is important for those critical organs located near the target. For example, it was observed that 8 of 20 patients for PTV margin‐based plans, the chest wall doses were above the constraint dose (at most 5 cc of the chest wall volume received 5 Gy)^10^ in this study, whereas all the chest wall doses calculated from robust optimization plans were within this dose–volume limit. Figure 4 shows an example of isodose distribution from a robust optimization plan (a) and from a PTV margin‐based plan (b) for one of the selected patients whose tumor target was located near the chest wall.

Archibald‐Heeren et al. 18 reported that tumors with large motion and large density variations in surrounding tissue may result in significant improvements in dose stability with robust optimization, whereas for smaller tumor motion of <1 cm, the effect was less significant. However, no correlation was found between ITV mean dose and tumor site/tumor size/tumor motion in this study. To subgroup the patients by tumor size, tumor location or tumor motion extent, more patient data might be needed to obtain meaningful statistical results. One last finding in this study was that PTV margin‐based plans delivered more MU compared to robust optimization plan. The median MU for PTV margin‐based plans was 479 MU (PTV‐IMRT) and 303 MU (PTV‐VMAT) higher compared to corresponding robust optimization plans (Table 2). This reduced number of MUs in robust optimization planning could translate into less treatment time, possibly less respiratory motion cycles during treatment.

## 5 CONCLUSION

Robust optimization plans provide robust target coverage for both ITV‐IMRT and ITV‐VMAT plans. The plan robustness was evaluated with perturbed doses by specifying a user defined shifted values from isocenter to ±3 mm in LR, ±5 mm in IS, and ±3 mm in AP directions. The perturbed doses of D_99_, D_98,_ and D_95_ were all satisfied at least 99% of the ITV to receive 95% of the prescription doses. In addition, better ITV dose coverage, better CI, and worse HI were found compared to PTV margin‐based plans. OAR doses were compared and the results showed that robust optimization plans have significantly reduced OAR doses especially for normal lung doses and OAR doses adjacent to the lung lesions. It was also observed that PTV margin‐based plans had higher MU compared to robust optimization plans.

## CONFLICT OF INTEREST

The authors declare no conflict of interest.
